# Mean glucose during ICU admission is related to mortality by a U-shaped curve in surgical and medical patients: a retrospective cohort study

**DOI:** 10.1186/cc9369

**Published:** 2010-12-10

**Authors:** Sarah E Siegelaar, Jeroen Hermanides, Heleen M Oudemans-van Straaten, Peter HJ van der Voort, Robert J Bosman, Durk F Zandstra, J Hans DeVries

**Affiliations:** 1 Department of Internal Medicine, Academic Medical Centre, Meibergdreef 9, 1105 AZ, Amsterdam, The Netherlands; 2 Department of Intensive Care Medicine, Onze Lieve Vrouwe Gasthuis, Oosterpark 9, 1091 AC, Amsterdam, The Netherlands

## Abstract

**Introduction:**

Lowering of hyperglycemia in the intensive care unit (ICU) is widely practiced. We investigated in which way glucose regulation, defined as mean glucose concentration during admission, is associated with ICU mortality in a medical and a surgical cohort.

**Methods:**

Retrospective database cohort study including patients admitted between January 2004 and December 2007 in a 20-bed medical/surgical ICU in a teaching hospital. Hyperglycemia was treated using a computerized algorithm targeting for glucose levels of 4.0-7.0 mmol/l. Five thousand eight hundred twenty-eight patients were eligible for analyses, of whom 1,339 patients had a medical and 4,489 had a surgical admission diagnosis.

**Results:**

The cohorts were subdivided in quintiles of increasing mean glucose. We examined the relation between these mean glucose strata and mortality. In both cohorts we observed the highest mortality in the lowest and highest strata. Logistic regression analysis adjusted for age, sex, Acute Physiology and Chronic Health Evaluation II (APACHE II) score, admission duration and occurrence of severe hypoglycemia showed that in the medical cohort mean glucose levels <6.7 mmol/l and >8.4 mmol/l and in the surgical cohort mean glucose levels < 7.0 mmol/l and >9.4 mmol/l were associated with significantly increased ICU mortality (OR 2.4-3.0 and 4.9-6.2, respectively). Limitations of the study were its retrospective design and possible incomplete correction for severity of disease.

**Conclusions:**

Mean overall glucose during ICU admission is related to mortality by a U-shaped curve in medical and surgical patients. In this cohort of patients a 'safe range' of mean glucose regulation might be defined approximately between 7.0 and 9.0 mmol/l.

## Introduction

Owing to inflammatory and neuro-endocrine derangements in critically ill patients, stress hyperglycemia associated with high hepatic glucose output and insulin resistance is common in the intensive care unit (ICU) [1]. This stress hyperglycemia is associated with poor outcome [2]. Moreover, several studies report a deleterious effect of glycemic variability over and above mean glucose after correction for severity of disease [3-6].

In 2001, van den Berghe and colleagues [7] published the first randomized controlled trial (RCT) comparing normalization of glycemia by intensive insulin treatment (IIT) with conventional glycemic control in a surgical ICU (glucose target: 4.4 to 6.1 mmol/L versus 10.0 to 11.1 mmol/L). The authors reported an impressive reduction in mortality with IIT. The same group failed to reproduce these findings in the entire population of patients in their medical ICU [8]; however, mortality was lower in the predefined subgroup of patients receiving IIT for more than 3 days. After the data were pooled from both RCTs, IIT seemed to be associated with a reduction in mortality [9]. On the basis of these 'Leuven trials', many hospitals decided to implement protocols and target normalization of glucose levels to improve patient care.

Recently, after the publication of two inconclusive multicenter studies (the Volume Substitution and Insulin Therapy in Severe Sepsis [VISEP] [10] and the GluControl [11,12] studies) followed by the NICE-SUGAR (Normoglycaemia in Intensive Care Evaluation-Survival Using Glucose Algorithm Regulation) trial [13], doubt was cast upon the benefits of tight glycemic control; the NICE-SUGAR trial investigators reported an absolute increase in deaths at 90 days with IIT (glucose target: 4.5 to 6.0 mmol/L versus 8.0 to 10.0 mmol/L). A recently published meta-analysis including this latter trial showed that IIT significantly increased the risk of hypoglycemia and conferred no overall mortality benefit among critically ill patients [14]. The goal of the present study is to report glucose and mortality data from cohorts of patients with a medical and a surgical admission diagnosis from a general ICU of a teaching hospital in The Netherlands.

## Materials and methods

### Cohorts, setting, and data collection

We collected information about patients admitted between January 2004 and December 2007 in a 20-bed medical/surgical ICU in a teaching hospital (Onze Lieve Vrouwe Gasthuis [OLVG], Amsterdam, The Netherlands) (the OLVG cohort). All data were anonymous and collected retrospectively, so no ethical approval was necessary. On average, one nurse took care of two patients, depending on the severity of disease. All beds were equipped with a clinical information system (MetaVision; *i*MD*soft*, Tel Aviv, Israel) from which all clinical and laboratory data were extracted. The glucose regulation algorithm was implemented successfully in 2001 [15], targeting for glucose values of between 4.0 and 7.0 mmol/L. The glucose protocol was started for every patient at the time of arrival at the ICU. Insulin infusion was started when admission blood glucose exceeded 7.0 mmol/L. When admission glucose was lower than 7.0 mmol/L, blood glucose was further measured every 2 hours and insulin was started when necessary (that is, when blood glucose exceeded 7.0 mmol/L). The nursing staff was instructed to use a dynamic computerized algorithm to adjust the insulin infusion rate, depending on the current glucose value and the rate of glucose change (based on the previous five measurements). The software also provided the time the next glucose measurement was due, which could vary from 15 minutes to 4 hours. Routinely, enteral feeding was started within 24 hours after admission, aiming at 1,500 kcal per 24 hours, and subsequently adjusted to the patient's requirements, except for the uncomplicated cardiac surgery patients, who do not receive enteral feeding if extubated within 24 hours. A duodenal feeding tube was inserted in case of persistent gastric retention. The tight glucose algorithm was deactivated when patients resumed normal eating.

We excluded readmissions, patients with a withholding care policy, and patients with only one glucose value measured during admission. From the clinical information system, we collected demographic variables, mortality rates in the ICU, and glucose values. For severity of disease measures, we used the Acute Physiology and Chronic Health Evaluation II (APACHE II) score [16]. Informed consent was not required according to Dutch Ethical Review Board regulations, because a retrospective analysis of anonymous data was performed.

### Glucose measures

For each patient, we calculated the mean overall glucose during admission from all glucose values measured during admission and the mean morning glucose from the first value available between 5 and 7 a.m. per patient per day. Glucose values mentioned in this paper stand for mean overall glucose unless stated otherwise. We calculated the standard deviation (SD) and the mean absolute glucose (MAG) change [6] per patient as markers of glycemic variability. Glucose was obtained from arterial blood samples by means of a handheld glucose measurement device (AccuChek; Roche/Hitachi, Basel, Switzerland). Results were automatically stored in the clinical information system.

### Data interpretation

The cohort characteristics are presented as mean ± SD or as median and interquartile range (IQR), depending on the distribution of the data. The mean glucose values and SDs were divided into five strata with equal numbers of patients per group. For each stratum, the ICU mortality was calculated. Subsequently, we performed a logistic regression analysis to calculate the odds ratio (OR) with 95% confidence intervals for ICU mortality per glucose stratum. The stratum with the lowest mortality incidence was used as a reference. In this model, we adjusted for age, sex, severity of disease (APACHE II score), occurrence of severe hypoglycemia (≤2.2 mmol/L), and admission duration (that is, ≤ or >24 hours). The last adjustment was done because glucose values are higher and have a wider range in the first 24 hours of admission, biasing the patients with longer admission times and corresponding lower mean glucose values. In a second model, adjustment for occurrence of mild hypoglycemia (≤4.7 mmol/L), which is also independently associated with mortality [17], was made.

## Results

In total, 5,828 patients were eligible for analyses of the mean glucose for the OLVG population after excluding 656 readmissions, 86 patients with a withholding care policy, and 160 patients with only one glucose value measured. This cohort consisted of 1,339 patients with a medical admission diagnosis (the 'medical' population) and 4,489 patients with a surgical admission diagnosis (the 'surgical' population). In the medical cohort, a median (IQR) of 34 (15 to 65) glucose values per patient were collected, and in the surgical cohort, a median (IQR) of 10 (5 to 14) values were collected. The median (IQR) admission durations were 64 (30 to 129) hours in the medical cohort and 22 (18 to 28) hours in the surgical cohort.

### Mean glucose

**Figure 1:**
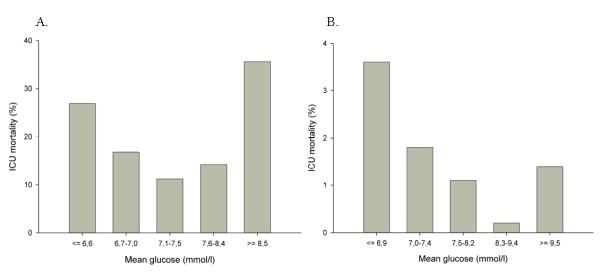
**Intensive care unit (ICU) mortality (y-axis) per mean glucose stratum (x-axis)**. **(a) **Medical population. **(b) **Surgical population.

**Figure 2:**
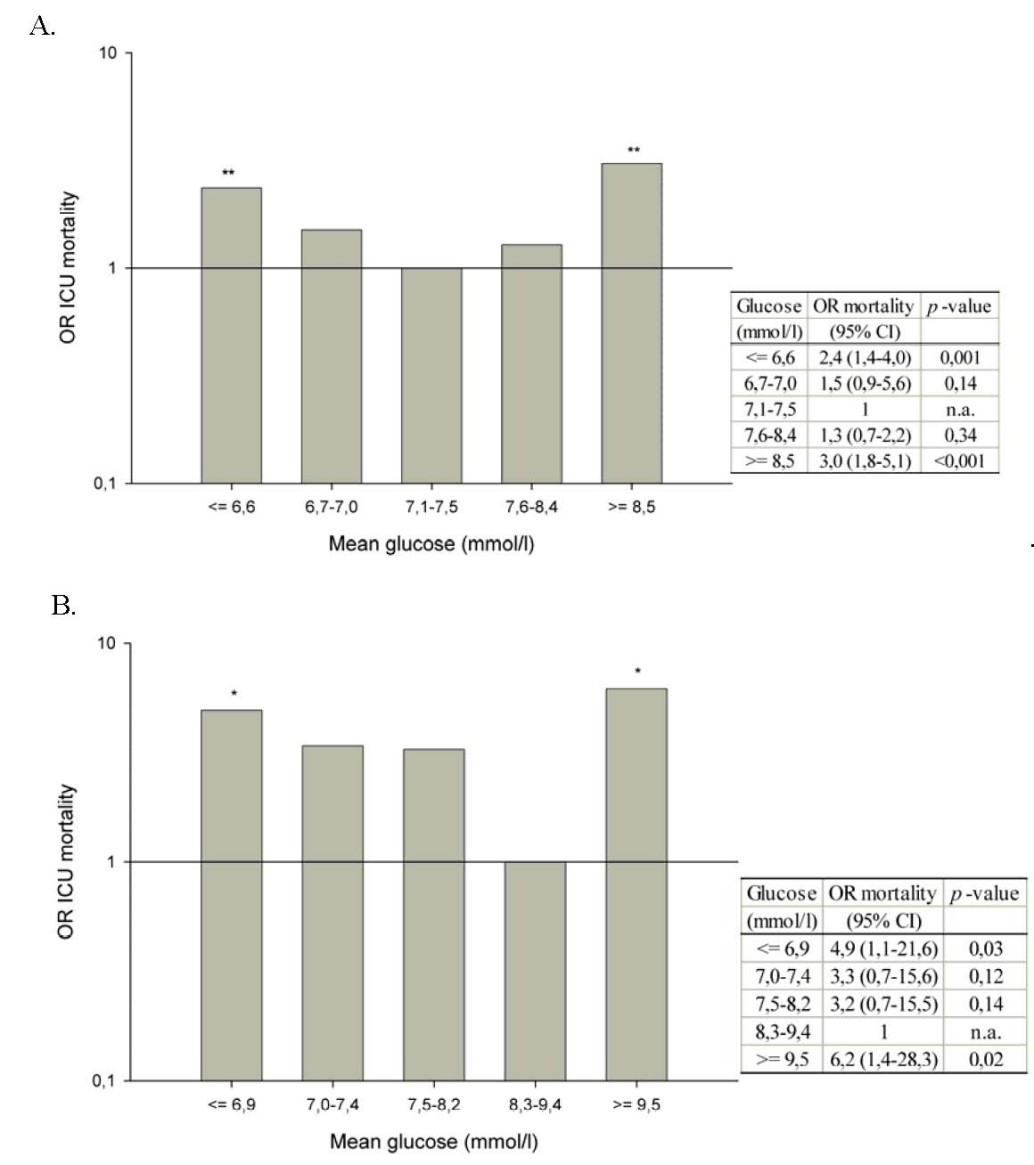
**Odds ratio (OR) for mortality (y-axis) per glucose stratum (x-axis) with the highest OR in the lowest and highest strata**. **(a) **Medical population. **(b) **Surgical population. Logistic regression model was adjusted for age, sex, APACHE II (Acute Physiology and Chronic Health Evaluation II) score, admission duration (≤ and >24 hours), and occurrence of severe hypoglycemia. *P < 0.05, **P < 0.001. CI, confidence interval.

The overall mean (SD) glucose values of the medical and surgical populations were 7.9 (2.7) and 8.1 (1.6) mmol/L, respectively (Table 1). The mean glucose values of the first 24 hours of admission were higher and had a wider range than did the mean glucose values after 24 hours (medical: mean [SD] 8.4 [3.3] mmol/L, range 3.7 to 40.2 mmol/L and 7.0 [1.4] mmol/L, range 3.2 to 31.1 mmol/L; surgical: mean [SD] 8.3 [1.9] mmol/L, range 0.6 to 27.5 mmol/L and 7.6 [1.7] mmol/L, range 3.2 to 15.7 mmol/L). The mean morning glucose values were 7.4 [2.6] mmol/L in the medical population and 7.7 [2.3] mmol/L in the surgical population. After division of the mean glucose of both populations into five equally sized strata, the lowest mean glucose stratum ranged from 6.7 mmol/L and lower in the medical cohort and from 7.0 mmol/L and lower in the surgical cohort. The highest stratum ranged from 8.5 mmol/L and higher in the medical cohort and from 9.5 mmol/L and higher in the surgical cohort. Mean glucose ranges per stratum and corresponding mortality rates per cohort are displayed in Figure 1. This results in a U-shaped curve relationship between mean glucose and mortality in both cohorts, with high ICU mortality in the lowest and highest glucose strata (medical: 26.9% and 35.6%; surgical: 3.6% and 1.4%). Logistic regression analysis showed that in both populations mean glucose values in the lowest and highest strata were associated with a significantly higher OR for ICU mortality compared with the stratum with the lowest mortality (Figure 2). This results in 'safe ranges' of 6.7 to 8.5 mmol/L in the medical cohort and 7.0 to 9.5 mmol/L in the surgical cohort. The non-linear U-shaped relationship between mean glucose and ICU mortality was supported by significance of the quadratic transformation of the mean glucose levels in this logistic regression model (*P *< 0.001). The characteristics of our populations, also subdivided in groups with low, 'safe range', and high glucose values, are displayed in Tables 1 and 2.

**Table 1:** Characteristics of the studied cohorts

	Medical population				Surgical population			
	Total *n *= 1,339	≤6.6 mmol/L *n *= 268	'Safe range' *n *= 804	≥8.5 mmol/L *n *= 267	Total *n *= 4,489	≤6.9 mmol/L *n *= 898	'Safe range' *n *= 2,694	≥9.5 mmol/L *n *= 897
Age in years, mean ± SD	61.8 ± 16.9	59.0 ± 18.4	62.5 ± 16.2	62.4 ± 17.0	66.0 ± 12.0	66.8 ± 12.5	65.4 ± 12.1	67.2 ± 11.3
Female gender, percentage	38.2	37.3	37.7	40.4	33.2	36.6	32.0	33.4
APACHE II score, mean ± SD	24.6 ± 8.8	24.8 ± 9.1	24.1 ± 8.1	25.8 ± 10.2	15.1 ± 4.6	16.3 ± 5.2	14.8 ± 4.5	14.7 ± 4.2
Diabetes mellitus, percentage	0.6	0.4	0.5	1.1	15.4	23.7	16.4	4.1
Died in the ICU, percentage	20.9	26.9	14.1	35.6	1.6	3.6	1.0	1.4
Died in the hospital, percentage	31.3	35.4	26.6	41.2	4.3	7.5	3.9	2.7
Morning glucose in mmol/L, mean ± SD	7.4 ± 2.6	5.9 ± 1.0	7.1 ± 1.2	10.3 ± 4.5	7.7 ± 2.3	5.8 ± 1.2	7.3 ± 1.7	10.6 ± 1.9
Overall glucose in mmol/L, mean ± SD	7.9 ± 2.7	6.0 ± 0.6	7.3 ± 0.5	11.6 ± 4.1	8.1 ± 1.6	6.4 ± 0.5	7.9 ± 0.7	10.7 ± 1.1
Hypoglycemia incidence, percentage	9.9	18.7	8.8	4.5	1.8	4.8	1.3	0.1
SD, median (IQR)	2.0 (1.5-2.9)	1.6 (1.2-1.9)	2.0 (1.6-2.6)	3.8 (2.7-5.4)	1.8 (1.3-2.3)	1.6 (1.3-2.0)	1.8 (1.4-2.4)	1.9 (1.4-2.6)
MAG change, median (IQR)	0.8 (0.5-1.1)	0.5 (0.3-0.8)	0.8 (0.6-1.0)	1.4 (0.9-2.0)	0.6 (0.4-0.8)	0.5 (0.4-0.7)	0.6 (0.4-0.9)	0.5 (0.3-0.7)
Caloric intake per 24 hours, mean ± SD	1,103.0 ± 758.4	1,159.3 ± 1,108.6	1,107.1 ± 507.2	1,033.6 ± 944.5	315.0 ± 392.3	427.7 ± 466.6	322.8 ± 387.5	181.5 ± 268.9
Use of insulin, percentage	88.5	79.5	93.3	82.8	64.0	93.1	71.8	11.6
Insulin dose in IU/hour, median (IQR)	1.4 (0.8-2.4)	0.6 (0.4-1.0)	1.4 (0.9-2.1)	3.4 (2.0-6.2)	1.2 (0.7-1.9)	1.0 (0.7-1.5)	1.3 (0.8-2.0)	1.5 (0.7-3.2)
Use of vasopressor drugs, percentage	86.0	19.4	11.8	15.4	94.8	94.1	94.2	97.0
Use of corticoids, percentage	92.5	91.0	94.8	86.9	99.1	99.0	99.1	99.1
Mechanical ventilation, percentage	81.6	81.7	85.0	71.2	97.9	97.3	97.9	98.6
CVVH, percentage	16.7	20.1	17.4	11.2	2.6	7.0	1.8	0.8

Characteristics of the studied cohorts are divided by mean glucose ranges. The 'safe range' refers to the mean glucose levels associated with the lowest mortality rates: 6.7 to 8.4 mmol/L in the medical cohort and 7.0 to 9.4 mmol/L in the surgical cohort. Hypoglycemia was defined as at least one glucose value of not more than 2.2 mmol/L. APACHE II, Acute Physiology and Chronic Health Evaluation II; CVVH, continuous veno-venous hemofiltration; ICU, intensive care unit; IQR, interquartile range; MAG, mean absolute glucose; SD, standard deviation.

**Table 2:** Percentage of patients per APACHE II admission category

	Medical population				Surgical population			
	Total *n *= 1,339	≤6.6 mmol/L *n *= 268	'Safe range' *n *= 804	≥8.5 mmol/L *n *= 267	Total *n *= 4,489	≤6.9 mmol/L *n *= 898	'Safe range' *n *= 2,694	≥9.5 mmol/L *n *= 897
Cardiovascular	18.0	11.6	19.9	18.7	88.2	81.0	88.3	95.1
Sepsis	16.5	22.8	16.0	11.6	1.2	2.8	1.0	0.1
After cardiac arrest	21.6	11.9	21.5	31.5	0.2	0.6	0.1	0.1
Gastrointestinal	4.3	4.1	4.2	4.9	5.3	8.7	5.0	2.8
Hematological	0.6	0.7	0.7	0	0.2	0.4	0.1	0.1
Renal	1.9	1.5	1.0	5.2	0.3	0.6	0.2	0.1
Metabolic	3.6	3.0	2.7	6.7	0.2	0.1	0.2	0.1
Neurological	11.5	18.3	10.3	8.2	0.9	1.1	1.0	0.3
Respiratory	22.0	26.1	23.5	13.1	3.6	4.8	4.0	1.2

The 'safe range' refers to the mean glucose levels associated with the lowest mortality rates: 6.7 to 8.4 mmol/L in the medical cohort and 7.0 to 9.4 mmol/L in the surgical cohort. APACHE II, Acute Physiology and Chronic Health Evaluation II.

### Other glycemic measures

**Figure 3:**
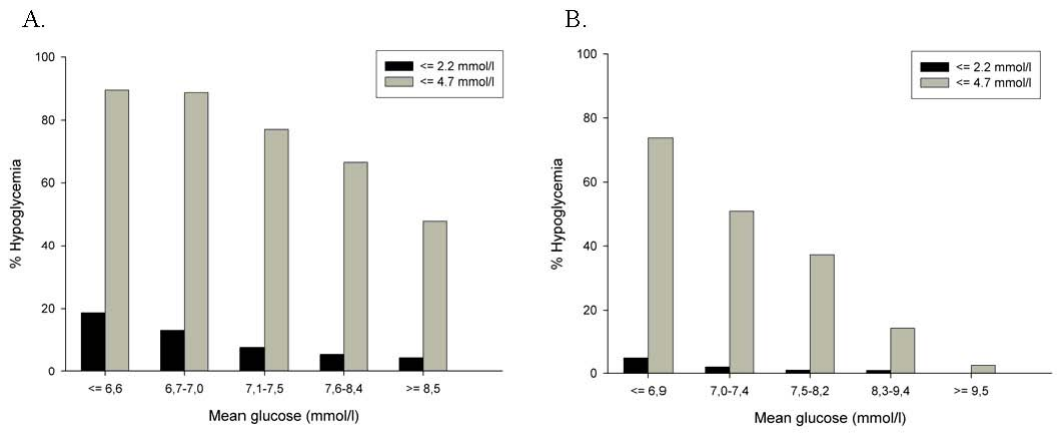
**Hypoglycemia incidence (y-axis) per mean glucose stratum (x-axis)**. **(a) **Medical population. **(b) **Surgical population. The y-axis represents the percentage of patients experiencing at least one severe (≤2.2 mmol/L, left bars) and mild (≤4.7 mmol/L, right bars) hypoglycemic event.

Overall, 9.9% and 1.8% of the medical and surgical patients, respectively, sustained at least one hypoglycemic episode, defined as a glucose value of not more than 2.2 mmol/L, during ICU admission. Seventeen point five percent of all deaths during ICU admission concerned patients who had experienced severe hypoglycemia (both groups). Twenty-eight percent of the patients who were in the lowest mean glucose strata and who died in the ICU experienced hypoglycemia, and 72% did not. The incidence of severe and mild (≤4.7 mmol/L) hypoglycemia in the different mean glucose strata is reported in Figure 3. When we adjusted the logistic regression model for occurrence of mild hypoglycemia with a cutoff value of 4.7 mmol/L, which is also independently associated with mortality [17], the OR (95% confidence interval) for ICU mortality in the lowest glucose stratum remained significant (medical: 2.6 [1.6 to 4.4], *P *< 0.001; surgical: 4.9 [1.1 to 22.1], *P *= 0.04).

In the medical cohort, glucose variability, both when expressed as the median of individual SDs and MAG changes [6], linearly increased with increasing glucose strata (SD median [IQR] 1.6 [1.2 to 1.9] to 3.8 [2.7 to 5.4] mmol/L, *P *for trend < 0.001; MAG 0.5 [0.3 to 0.8] to 1.4 [0.9 to 2.0] mmol/L per hour, *P *for trend 0.007). However, in the surgical cohort, no consistent trend in glucose variability across the glucose strata was seen (SD median [IQR] 1.8 [1.3 to 2.3] mmol/L; MAG 0.6 [0.4 to 0.8] mmol/L per hour). Adjusting the logistic regression model for variability did not change the above-described relationship between mean glucose and mortality (data not shown).

## Discussion

The salient finding of this investigation is that in this mixed medical and surgical cohort of critically ill patients, mean glucose values of between approximately 7.0 and 9.0 mmol/L during ICU stay were associated with the lowest OR for ICU mortality, whereas mean values of below 7.0 and greater than 9.0 mmol/L confer significantly higher ORs. These results were attained while using a dynamic glucose algorithm that targeted for glucose values of between 4.0 and 7.0 mmol/L. The finding that hyperglycemia is associated with increased mortality is in accordance with published literature [2,18,19]. Also, the U-shaped curve we found, with increased mortality in the lower and upper parts, is described earlier in patients with myocardial infarction during admission [20-22], more generally in patients with type 2 diabetes mellitus [23], and in the ICU setting [24-26], corroborating this finding. The optimum glucose levels in the ICU setting reported previously are somewhat lower than we found. This is possibly due to differences in inclusion criteria or uncertainty about the practice of tight glycemic control [26], lack of regression analysis between the strata [25], or a different method to assess mean glucose [24]. Another difference between our and other ICU cohorts is the high percentage of patients admitted after cardiac arrest (Table 2), a population with a high mortality rate. Also, the percentage of patients with diabetes in our cohort might be underestimated since we scored diabetes only when the patient used anti-hyperglycemic drugs. However, how these factors might influence the position of the U-curve in relation to the x-axis is not known.

Hypoglycemia is associated with increased risk of ICU and hospital mortality [17,27-29]. In our population, the incidence of hypoglycemia was highest in the lowest mean glucose cohorts in which mortality was higher as well. In addition, a significant percentage of the patients who died had experienced a hypoglycemic episode. However, hypoglycemia can account only partially for the high mortality rate in the lowest mean overall glucose stratum since 72.0% of the non-survivors did not experience severe hypoglycemia. Also, when the logistic regression model was adjusted for occurrence of severe or mild hypoglycemia, the OR for mortality remained significantly higher for those patients with a mean glucose in the lowest quintile. However, it might be possible that some hypoglycemic episodes were not recorded because of intermittent sampling, or were underestimated because of the AccuChek point-of-care meter used for glucose measurements, the results of which tend to be higher than those obtained from the laboratory [30,31]. Therefore, the contribution of hypoglycemia to ICU death could be underestimated and needs further research using continuous glucose measurement. An alternative explanation for increased mortality at lower glucose values might be that tissues with insulin-independent glucose uptake may suffer from insufficient glucose availability at lower concentrations. In our cohort, glucose variability increased with increasing glucose strata in the medical cohort. In the surgical cohort, no consistent relationship was found. Since glucose variability is associated with mortality [6], it is unlikely that this contributes to the higher mortality in the lower glucose strata.

In the NICE-SUGAR study, the mean glucose of the IIT group (6.4 mmol/L) falls into the stratum with increased mortality compared with the conventional group (8.0 mmol/L), which lies in the safe range of both OLVG populations (Figure 1) [13]. Thus, the findings of the NICE-SUGAR trial are in accordance with the mortality data from our cohort. This is in contrast to the data of both Leuven studies. The means of the IIT groups of both the Leuven studies (6.1 mmol/L in the medical population [8] and 5.7 mmol/L in the surgical population [7]) fall into the lowest mean glucose stratum in the corresponding OLVG cohorts, in which mortality is highest. The means of the conventional groups in the Leuven studies (8.5 mmol/L in the medical as well as in the surgical population [7,8]) lie in the safe ranges of both OLVG populations (Figure 1).

A possible explanation for the low mortality of the Leuven IIT group might be the way of feeding. In a recent paper, Marik and Preiser [32] suggested that the use of intravenous calories could explain differences between populations treated with IIT, with a positive effect of IIT in patients who receive most of their calories intravenously. In our population, as opposed to the Leuven studies, only 0.7% of carbohydrates were given parenterally. In populations predominantly fed parenterally, the relationship between mean overall glucose and mortality might be different. Also, glycemic swings are a known risk factor of ICU death and might contribute to differences in mortality rate [4,5]. However, it is unlikely that differences in glucose variability explain the higher mortality in our cohort compared with the Leuven IIT group as the medians (IQR) of the individual median SDs are roughly comparable (Leuven medical 1.99 [1.57 to 2.66] mmol/L [33] and OLVG medical 2.03 [1.54 to 2.86] mmol/L). In addition, other explanations have been proposed to explain the diverging outcomes of Leuven and NICE-SUGAR [34].

The mean glucose of the OLVG population (medical: 7.9 mmol/L; surgical: 8.1 mmol/L) was higher than the target range, which was between 4.0 and 7.0 mmol/L. Other studies of IIT also did not reach their target range, illustrating the difficult implementation of this therapy [10,12,13]. The high percentage of corticosteroid treatment in our population might have contributed (Table 1). Also, the relatively short ICU duration of stay in the predominantly surgical population of the OLVG explains that mean glucose is slightly higher than the target (median ICU stay was 22 hours in our cohort compared with 3 days in the Leuven cohort and 4.2 days 'on algorithm' in the NICE-SUGAR study) because of the time needed to reach target. Glucose values were indeed higher and had a wider range in the first 24 hours of admission. Furthermore, our patients were treated in a normal-care setting without the extra stimuli of a trial setting to achieve the target. It should be noted that mean glucose does not equal time in target range, since the protocol requires more frequent sampling when not in target, thus falsely inflating the mean.

In our logistic regression model, we adjusted for severity of disease and admission duration less or more than 24 hours since both high and low glucose levels could be a manifestation, rather than a cause, of severe disease. Glucose values are higher and have a wider range in the first 24 hours of admission, biasing the patients with longer admission times and corresponding lower mean glucose values. A limitation of our correction for severity of disease is the use of the APACHE II score, because the use of APACHE II score to predict mortality is not validated for cardiac surgery patients. However, this adjustment is the best available method [35].

## Conclusions

In our mixed cohort of surgical and medical patients, the mean glucose during ICU stay was related to mortality by a U-shaped curve; a 'safe range' for mean glucose can be defined as between approximately 7.0 and 9.0 mmol/L, while both higher and lower mean values are associated with higher mortality. This finding applied to the surgical as well as the medical patients. Hypoglycemia seems to only partially explain the high mortality rate in the lowest mean glucose quintile, and glucose variability does not. Second, comparison of the combined Leuven, NICE-SUGAR, and our cohorts demonstrates that the increased mortality in the IIT group of NICE-SUGAR is in line with our U-shaped curve but that the low mortality in the intensively treated Leuven group is not. The percentage of calories given parenterally may influence the relationship between mean glucose and mortality. We await further studies, but according to these findings, we recommend treating hyperglycemia in the ICU in a moderately intensive way in both medical and surgical patients, targeting for mean glucose values of between approximately 7.0 and 9.0 mmol/L and avoiding hypoglycemia. This 'safe range' should be studied prospectively in randomized clinical trials.

## Key messages

• During ICU admission, mean glucose relates to mortality by a U-shaped curve.

• A mean glucose range of 7.0 to 9.0 mmol/L is associated with the lowest mortality in our cohort.

• Occurrence of hypoglycemia does not fully explain the high mortality in the lower glucose strata.

## Abbreviations

APACHE II: Acute Physiology and Chronic Health Evaluation II; ICU: intensive care unit; IIT: intensive insulin treatment; IQR: interquartile range; MAG: mean absolute glucose; NICE-SUGAR: Normoglycaemia in Intensive Care Evaluation-Survival Using Glucose Algorithm Regulation; OLVG: Onze Lieve Vrouwe Gasthuis (hospital); OR: odds ratio; RCT: randomized controlled trial; SD: standard deviation.

## Competing interests

The authors declare that they have no competing interests.

## Authors' contributions

SES and JH participated in the design of the study, performed the statistical analysis, and wrote the manuscript. HMO-vS, PHJvdV, and DFZ participated in the design of the study, contributed to the interpretation of the data, and revised the manuscript critically for important intellectual content. RJB participated in the design of the study, performed acquisition of the data, contributed to the interpretation of the data, and revised the manuscript for important intellectual content. JHD participated in the design of the study, contributed to the interpretation of the data, and participated in the writing of the manuscript. All authors read and approved the final manuscript.
